# Dynamic Capabilities and an ESG Strategy for Sustainable Management Performance

**DOI:** 10.3389/fpsyg.2022.887776

**Published:** 2022-05-26

**Authors:** Yi Liang, Min Jae Lee, Jin Sup Jung

**Affiliations:** ^1^School of Foreign Languages, Neijiang Normal University, Neijiang, China; ^2^Department of International Trade and Logistics, Mokwon University, Daejeon, South Korea; ^3^Department of International Business, Chungbuk National University, Cheongju, South Korea

**Keywords:** dynamic capabilities, absorptive capability, adaptive capability, ESG strategy, sustainable management performance

## Abstract

This research explores the dynamic capabilities required for firms to implement environmental, social, and governance (ESG) strategies, and investigates sustainable management performance that can be created based on them. By using dynamic capabilities theory, we integrate sustainable management and the ESG literature to suggest a research model and identify the factors that act as the catalysts achieving sustainability. The data used for the analysis were collected from 78 firms listed on the Korea Exchange (KRX) with assets totaling more than 2 trillion Korean won. In this study, the partial least squares structural equation model (PLS-SEM) is applied. We found that absorptive capability and adaptive capability significantly affect sustainable management performance through implementation of the ESG strategy as a mediating variable. In particular, a firm’s implementation of an ESG strategy is a significant determinant that impacts sustainable management performance. We also believe our model contributes to the current knowledge by filling several research gaps, and our findings offer valuable and practical implications not only for achieving sustainable growth but also for creation of competitive advantage.

## Introduction

We often witness the rise and fall of firms due to various environmental changes (technological evolution, pandemics, etc.). In other words, firms that appropriately respond to changes in the business environment get an opportunity to maintain their business activities or prosper, while those that fail to adapt can lose their competitive advantage and face expulsion from the market. In particular, the COVID-19 pandemic further increases uncertainties in the business environment, triggering innovations for firms to survive. In this situation, firms seek solutions by establishing and implementing various strategies, such as changing business models or restructuring to enhance their sustainability in order to survive.

Discussions have steadily taken place in academia on ways to increase corporate sustainability. This stream of research assumes the resource heterogeneity and stability of the strategic resources over time. Some researchers, including Barney (1991), argued that the relationship between a firm’s resources and sustained competitive advantage is possible if the resources are valuable, rare, inimitable, non-sustainable, and organized (Pisano, 1994; Grant, 1996; Eisenhardt and Martin, 2000; Bhandari et al., 2020). The resource-based view (RBV) focuses on the internal strengths and weaknesses of the firm, as opposed to the external environmental model of competitive advantage, which emphasizes on opportunities and threats (Bhandari et al., 2022). On the other hand, climate science enthusiasts and environmental economists have emphasized that firms need to address the imbalance between demand and natural supply if they want to be sustainable and achieve the United Nations’ Sustainable Development Goals (SDGs; Dasgupta, 2021). Therefore, scholars have been raising the need for stakeholder governance (Barney, 2018; Amis et al., 2020; Barney et al., 2021) to correct the supply–demand imbalance of resource depletion. They point out that attention should be paid to reducing their demand for environmental, social, and governance-related (ESG) footprints and helping to sustain their supply capacity.

Recent studies on these discussions emphasize the harmonious development of the economy, society, and the environment to achieve corporate sustainability (Dey et al., 2020; Henderson, 2021; Alkaraan et al., 2022). Previously, the literature mainly focused on economic (or financial) performance when predicting a firm’s sustainability. Lately, however, there has been consensus on the argument that sustainability can be improved when firms coexist with society (Ben-Amar et al., 2017; Holden et al., 2017). These discussions initially developed around international organizations (e.g., the UN, the OECD, and the World Bank), but have now expanded to include the private sector. Meanwhile, a number of studies examining this have shown that firms can benefit financially when they address environmental or societal concerns, but dismiss situations in which environmental and social aspects cannot be aligned with financial performances (Hahn et al., 2015). Accordingly, there is an increasing need for firms to understand their impact on society and the environment through non-financial performance (Schaltegger and Hörisch, 2017). When evaluating this non-financial performance, the environmental, social, and governance (ESG) framework is the one most commonly used and has emerged as a new measure for predicting corporate sustainability. Note that the ESG framework was initially used as an indicator to measure non-financial performance when investors (or asset management institutions) make investment decisions.

However, the ESG concept has recently been recognized as an essential management strategy for the survival of firms. There is a growing trend worldwide for firms to voluntarily disclose ESG information using standards and frameworks presented by the Global Reporting Initiative (GRI) and the Sustainability Accounting Standards Board (SASB). As such, ESG activities have become a trend for sustainable growth, but it is also true that many firms are unable to respond to these changes. For example, in South Korea (hereafter, Korea), the government encourages firms to engage in ESG activities, but only some corporations respond systematically to these changes, and most firms are not even discussing it. In addition, in order to achieve the lofty goal of sustained competitive advantage, the majority of Korean firms have profit maximization as their objective at the cost of ESG degradation. Therefore, for many Korean firms to continue to achieve competitive advantage without falling behind in the global market, it is necessary to identify the strategic approach that allow firms to respond quickly to environmental changes and seek effective management strategies for sustainability.

Achieving a competitive advantage is a strategic approach that is being pursued by all competitors in parallel. When a firm has a sustained competitive advantage, the strategic approach is to create value that belongs only to that firm, where imitation is not possible (Barney, 1991, 2001; Bhandari et al., 2020; Barney et al., 2021). With the rapidly changing business environment, there is an increasing interest in how to create unique value. The dynamic capabilities (DCs) perspective is actively used as a theoretical framework in this vein. Therefore, the focus of our research is on the firm’s DCs and how they create sustained competitive advantage.

The DCs perspective explains that a firm can achieve a sustainable competitive advantage by reconfiguring various resources and capabilities according to the changing environment (Teece et al., 1997). Previous studies have shown that a firm develops DCs through three standard features and processes that directly or indirectly affect its competitive advantage. The first factor is *absorptive capability*, which recognizes new-value external information, assimilates it, and applies it to commercial ends (Cohen and Levinthal, 1990). The second factor is *adaptive capability*, which is defined as a firm’s ability to identify and capitalize on new market opportunities (Miles et al., 1978; Chakravarthy, 1982; Hooley et al., 1992). The third factor is *innovative capability*, which refers to a firm’s ability to develop new products and/or markets by aligning an innovative, strategic orientation with innovative behaviors and processes (Wang and Ahmed, 2004, 2007).

Meanwhile, all these studies focus on creating financial value in achieving a competitive advantage based on DCs. However, a severe problem still resides in the fact that all three of these approaches (i.e., DCs) are experiencing a lack of sustainable social value that modern society is recently aiming for. In other words, although a firm’s competitive advantage desperately needs the creation of sustainable social value, the extant literature tends to shed light only on economic value, such as corporate growth and increases in sales. In particular, firms should be aware of the value of social responsibility and should meet the ethical demands of stakeholders because they are strategically crucial to improving a firm’s long-term performance. In this vein, no one can guarantee that a firm will be a long-lived organization if it does not appropriately assess the importance of sustainable social value, which indicates the necessity for a study dealing with cementing the gap between extant theoretical discussions and reality.

Based on the series of discussions above, we suggest several research objectives. First, we would like to provide a foundation for understanding the capabilities and structures to achieve competitive advantage by creating sustainable management performance (SMP) using ESG strategies. The DCs-related studies mentioned above have developed around a firm’s financial performance, making it somewhat challenging to understand the management trends that have been changing due to recent social and environmental value considerations. In other words, as these values emerge as a critical factor influencing corporate sustainability, the activities to find capabilities to create them are accelerating around various firms. In particular, we argue that it is necessary to discuss the implementation of an ESG strategy as a mediating variable for achieving SMP based on social and environmental values as well as financial values. Second, to the best of our knowledge, we have not seen an empirical analysis successful exploring how to improve a firm’s SMP by setting an ESG framework as a strategic process. Porter and Kramer (2019) confront the firm’s sustainability and ESG literature at the firm level through the concept of “shared value.” However, attempts to measure the “shared value” have not been very successful, even after Porter et al.’s (2011) contribution in this direction. Extant research has applied survey data or archival proxies in strategic management in general terms, with the latter predominating in highly cited contributions. We will design a research model suitable for measuring ESG and we will prove it through empirical analysis for firms that are pursuing a strategic approach for actual ESG implementation.

The potential expected effects of this study and the subsequent contributions are as follows. First, we propose an integrated framework to realize sustainable values or address social and environmental problems, such as development- and pollution-caused polarization, as well as economic value from the sustainable management perspective. Such a framework will help minimize the gap between extant theoretical discussions and reality and will set a direction to improve corporate sustainability. Second, we present the DCs and the ESG strategy needed for firms eager to achieve sustainable development, significantly providing the direction and foundation necessary to implement the ESG strategy.

## Theoretical Background and Hypothesis Development

The theoretical basis of our analysis is DCs perspective. DCs theory provides the understanding of the critical role of firm’s capabilities and their changes in shaping organizational behavior and performance (Teece et al., 1997; Teece, 2007; Wang et al., 2015). The core idea of this DCs theory is that in order to develop core competencies for competitive advantage in a rapidly changing business environment, firms should integrate, nurture, and reorganize internal and external resources in response to environmental changes (Teece et al., 1997). DCs theorists, including Teece et al. (1997), argue that through this framework, firms can understand the importance of innovation and achieve long-term surviving. This theory aims to examine sustainable growth methods based on firm’s capabilities and excellent strategies, while also containing innovation rather than just accepting the status quo of business environment. According to this perspective, firm’s decision-making process, behavior, and strategic response are primarily shaped by the rationale for achieving competitive advantage and restructuring the business environment. Competitive advantage is ensured for long-term competitiveness by generating sustainable management performance (SMP) in a constantly changing business environment (Barney et al., 2021; Bhandari et al., 2022). To achieve competitive advantage, firms typically strive to reduce uncertainty and improve performance through their strategies to meet the expectations of their stakeholders (Freeman et al., 2021). In the same vein, Gueler and Schneider (2021) posited that firms can achieve SMP by constantly supplementing and changing capabilities according to the needs of stakeholders. From this perspective, we argue that competitive advantage can be achieved by developing and fostering the DCs for firms to respond to changing environments. Specifically, the recent business environment requires a paradigm shift to coexist with stakeholders rather than prioritizing shareholder interests (Henderson, 2021). For this reason, many firms are expected to attempt to integrate and coordinate internal and external resources to acquire their DCs to meet the expectations of stakeholders. In particular, the movement to redefine the core values, strategies, and structures of firms is spreading as the social demand for a shift to stakeholder capitalism increases. In addition, firms will strive to improve SMP in a changing business environment by establishing strategies that are considered socially and institutionally appropriate based on these DCs. Therefore, unlike previous studies that primarily considered exploring the relationship between a firm’s own resources and its financial performance, we are interested in examining the DCs and ESG strategy of firms to achieve significant SMP to enhance their competitive advantage.

### Dynamic Capabilities and Sustainable Management Performance

Recently, competition between firms has intensified with the deepening uncertainty in the business environment. Accordingly, it not easy to guarantee a firm’s survival with existing strategic thinking that only seeks solutions based on its core resources and assets. Many scholars, including Teece et al. (1997), argued that DCs are needed for a firm’s survival and prosperity in a rapidly changing environment, through which the CEO can gain an innovative perspective to secure long-term competitiveness (Eisenhardt and Martin, 2000; Rindova and Kotha, 2001; Zollo and Winter, 2002; Teece, 2007).

Specifically, Tallman (1991) and Luo (2002) highlighted DCs as a source that enables MNCs to achieve a sustainable competitive advantage in the global market. According to Uhlenbruck et al. (2003) and Cepeda and Vera (2007), DCs develop strategies necessary to maintain a long-term competitive advantage in a highly uncertain and changing environment, enabling them to cope with crises occurring in a business environment. It has been widely agreed that there is a direct and positive relationship between DCs and a firm’s performance (Wang and Ahmed, 2007; Wilden et al., 2013; Wilhelm et al., 2015; Girod and Whittington, 2017). Meanwhile, some studies showed that DCs do not guarantee successful results for firms (Eisenhardt and Martin, 2000; Zahra et al., 2006). However, the existing literature has focused on the financial aspects of corporate performance due to DCs and does not deal with how it can affect the sustainable (including social and environmental) performance recently required by society (see Appendix 1).

As we all know, firms’ business environments have more volatility, complexity, uncertainty, and ambiguity than before (Teece, 2018). In particular, advances in technology (e.g., the Fourth Industrial Revolution) and the COVID-19 pandemic are accelerating these changes in the business environment. As uncertainty in corporate management grows, a firm’s competitive advantage is focused on sustainability rather than economic (or financial) growth. In this vein, many firms have recently shifted their operational objectives to a direction that increases sustainability. For example, firms such as Apple, Amazon, and GM are revising their strategies to meet the needs of stakeholders and secure capabilities to achieve a competitive advantage, breaking away from the existing strategic framework that strives to maximize shareholder profits.^1^

Focusing on this stakeholder capitalism perspective, scholars are increasingly discussing how firms should cover not only financial performance but also social and environmental value creation in order to improve sustainability (Henderson, 2021). Hussain et al. (2018) highlighted how a firm’s social responsibility activities could eventually improve shareholder profits. Kanashiro and Rivera (2019) explained that firms should shift their management policies from economic performance oriented to sustainable management that emphasizes environmental management and social responsibility at the same time. Therefore, it is essential for firms to secure DCs that help improve sustainability in order to effectively and innovatively change existing lagging operational systems according to volatile business trends.

In a similar vein, Cohen and Levinthal (1990) and Zahra and George (2002) pointed out that the more dynamic the business environment, the more critical the absorptive capability to improve sustainability. Absorption capacity refers to an organization’s ability to acquire, absorb, and use new information and knowledge (Cohen and Levinthal, 1990; Reinhardt, 1998). An absorptive capability provides a platform for generating sustainability-oriented learning, which in turn encourages organizations to adopt the necessary behaviors in response to sustainability situations and opportunities (Todorova and Durisin, 2007). Therefore, Lichtenthaler (2009) highlighted the importance of absorbing market knowledge for a firm’s sustainable growth. Moreover, Bhupendra and Sangle (2017) showed that an absorptive capability helps build a strong reputation, and gives legitimacy to the firm’s activities through sustainable strategies and knowledge management, which creates differential advantages and improves performance in the market. That is, deeper learning and dynamic awareness of stakeholder preferences through absorption capabilities can help a firm create solid growth in a future market.

Meanwhile, Oktemgil and Greenley (1997) argued that firms should be based on adaptive capabilities to achieve sustainable performance. In particular, an adaptive capacity is increasingly recognized as a critical attribute of environmental management. Tuominen et al. (2004) found that firms with an adaptive capacity create innovations that benefit not only financial performance but also social equity and conservation of the environment. Wong (2013) pointed out that the adaptive capabilities of firms in environmental management are critical organizational capabilities that are valuable to sustainable performance. In particular, as interest in environmental issues such as climate change has grown recently, strengthening an adaptive capability to environmental transformation is emerging as a very important competency for corporate sustainability.

As such, the literature reveals that absorptive and adaptive capabilities lead to sustainable performance improvement. In order to create SMP, including for society and the environment, the presence or absence of DCs to adapt and lead changing management trends can be a crucial factor. Based on the previous arguments, we propose the following hypotheses.

*H.1-1 An absorptive capability will positively affect the creation of sustainable management performance.*

*H.1-2 An adaptive capability will positively affect the creation of sustainable management performance.*

### The Mediating Effect of an ESG Strategy

No one will object to the argument that strategy is a key factor influencing the sustainable growth of a firm. That is because a successful strategy guarantees a firm’s prosperity, but a failed strategy can bring disaster. In this vein, Teece (2007, 2012) explained that firms could seek effective strategies to respond to environmental changes based on DCs linked to the development of competitive advantage. Ringov (2017) emphasized that firms create value if they develop and implement a suitable strategy based on their resources and capabilities (i.e., operational and dynamic capabilities). As such, firms can achieve superior performance if DCs underpin their strategies.

In this research, an ESG strategy, as one of the critical determinants of sustainable growth, was chosen in order to examine its mediating effect on the link between DCs and a firm’s SMP, because more firms are seeking strategies in terms of ESG to improve sustainability. According to the changing business environment, firms are considering their roles and responsibilities in order to secure sustainability beyond simply pursuing profits, and there is a movement to redesign the existing management systems based on an ESG strategy (Van Duuren et al., 2016). For example, Microsoft established a strategy to achieve a carbon-negative footprint by 2030 (i.e., carbon absorption is to be higher than carbon emissions) and is actively participating in solving climate problems. Netflix set inclusion as a corporate cultural value in 2017, revealed the gender and racial proportions of its employees, and is increasing recruitment of Hispanic and Latino talent. SK has increased the board of directors’ independence, and strengthened management monitoring and check functions by separating the roles of CEO and chairman of the board of directors to enhance the trust of stakeholders. As such, numerous firms are looking for ways to effectively allocate their resources on ESG, checking whether firms are realizing social functions and moving away from existing management strategies focused on profits maximization.

Meanwhile, to establish a successful overall strategy for a firm, it is necessary to focus core competencies on the ESG strategy. In particular, in any strategic management process, DCs should be taken into consideration to enable businesses to achieve their ESG goals. Amit and Schoemaker (1993) argued that DCs enable firms to develop strategies necessary to maintain long-term competitive advantages in highly uncertain and changing environments, and such strategies enable firms to respond well to crises occurring in competitive environments. Therefore, we argue that DCs create sustainable value if they positively contribute to the development and support of an ESG strategy.

The literature shows there is no doubt about the positive contribution of an ESG strategy to value creation (Wang and Ahmed, 2007; Parnell, 2011). Indeed, firms develop ESG strategies to create value for their stakeholders. However, different empirical studies postulate various relationships among firms’ strategies, DCs, and performance or value (Parnell et al., 2015; Yi et al., 2015; Rashidirad et al., 2017). We propose that sustainable value creation may not be perfectly accessible if DCs do not foster the firm’s ESG strategy. For instance, one of the primary sources of value in an ESG strategy is to develop long-term relationships with stakeholders for coexistence and co-prosperity.

**Figure 1:**
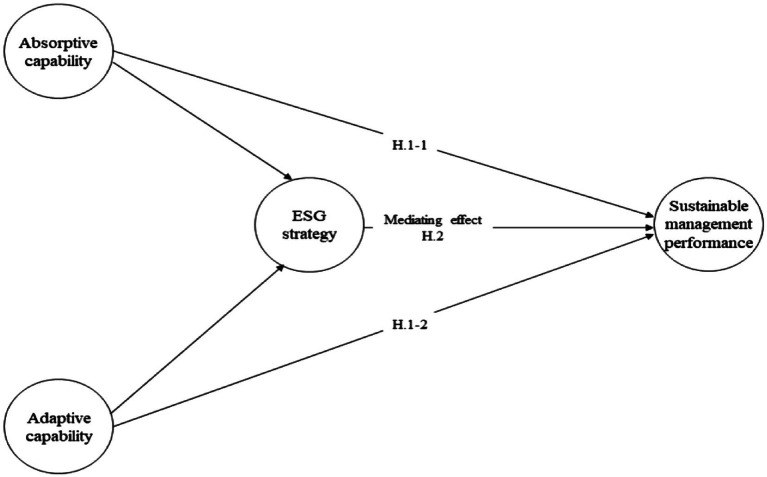
Research model.

We posit that such goals can be achieved if a firm develops a strategy to improve a shareholder capitalism system that is fostered by their DCs. Thus, ESG strategies will assist firms in deploying DCs that achieve SMP (see Figure 1).

*H2. An ESG strategy mediates the positive effect of dynamic capabilities (absorptive and adaptive capabilities) on a firm’s sustainable management performance*.

## Methodology

### Sample and Data Collection

The population for this study was firms with total assets of 2 trillion Korean won or more and that were listed on the Korea Exchange (KRX) as of December 2020. In order to promote ESG activities, the Korean government has made it mandatory for ESG activities (e.g., environmental information, sustainability, and firm governance) to be disclosed by firms with total assets of more than 2 trillion won as of 2021. The standard for disclosure is total assets, and the Korean government plans to reduce the standard amount every year to expand the number of firms subject to disclosure. For example, governance of disclosure will expand to firms with total assets of more than 1 trillion won in 2022, dropping to assets totaling more than 500 billion won in 2024, and governance will apply to all KRX-listed firms from 2026 on. For 2021, 215 firms were subject to disclosure, and this study surveyed them to collect data.

For the survey, each firm’s website was visited and checked for the email address of the ESG manager. However, one problem revealed at this time was that many firms did not disclose email addresses and contact names of ESG managers on the website. For this reason, helped by PhD students, there was no choice but to call as many firms as possible to inquire about the email address of the ESG manager, and firms that did not provide that email address were asked for the contact information of an investor relations (IR) representative. We selected senior managers who responsible of ESG, such as chief executive, vice president, senior director, and general managers in each firm to serve as key informant. The survey was assigned one person per firm, who have a wide knowledge of firm’s capabilities and ESG strategy. In particular, several firms were piloted to have representation of the firm. As a result of such pilot tests, some validity was obtained for these processes.

Building on the previous literature (Wang and Ahmed, 2007; Abdul-Rashid et al., 2017; Dey et al., 2020), we used Korean questionnaires to collect data for this research. The questionnaire was modified and written appropriately for this study based on items used in a previous study. Once the draft questionnaire was developed, we got feedback from several academic and managerial experts. Feedback from these experts was then taken and integrated into the final questionnaire. Several Korean professors and managers were invited to check whether the questionnaire was precise. A few minor changes were applied to increase clarity based on their feedback.

Before conducting the survey, a pilot test was undertaken to check the appropriateness of each question. Five ESG managers from SK and POSCO, and professors studying ESG strategies of Korean firms participated in the preliminary investigation. They confirmed that most of the questions were easily understood, but they also advised us to replace some words with better terms. Their suggestions were reflected in the final version. The finalized questionnaire was emailed to the firm’s ESG managers and investor relations representatives; emails sent to IR managers included a request to deliver the questionnaire to ESG-related departments and managers.

The survey was conducted over the 2 months from November to December 2021. At the end of that time, 80 questionnaires were finally collected, of which 78 were used for the final analysis because two were missing too much information (a response rate of 36.27%).

### Variable Measurement

The dependent variable in this study was the firm’s SMP combining economic, environmental, and social performance (Abdul-Rashid et al., 2017). To measure this, we inquired about (1) financial and market-based performance, (2) social performance, and (3) environmental performance. A firm’s financial and market-based performance was evaluated by modifying the measurement factors recommended by Panigyrakis and Theodoridis (2007). Specifically, we included three items: growth in sales, growth in profitability (He and Wong, 2004; Lumpkin and Dess, 2006), and growth in market share (Wang and Ahmed, 2007). The firm’s social and environmental performances were evaluated by referring to measurement factors used in Dey et al. (2020), including three additional items in each. A firm’s social performance alleviates inequality, strengthens social safety, and solves social problems. The firm’s environmental performance reduces carbon emissions, reduces resource usage, and improves the environment. These items were designed based on Zhu et al. (2008), Adebanjo et al. (2016), Abdul-Rashid et al. (2017), and Inman and Green (2018). Each item was rated on a five-point Likert scale ranging from 1 (much worse) to 5 (much better) by comparing the firm’s performance to its competitors over the previous 5 years.

The independent variable in this study was DCs. This study adopted the item scale of García-Morales et al. (2008), which was based on the definition of absorptive capability by Cohen and Levinthal (1990). Moreover, this study referred to items used to measure adaptive capability as defined by Gibson and Birkinshaw (2004) and Wang and Ahmed (2004). Each item was rated on a five-point Likert scale ranging from 1 (strongly disagree) to 5 (strongly agree; see Appendix 2 for details).

The mediation variable in this study was ESG strategy. This study included questions based on the K-ESG index developed by the Ministry of Trade, Industry, and Energy in 2021. Specifically, it requested four items for each strategy implementation. It asked about the establishment of environmental management strategies and action plans, management of environmental business performance, and support for stakeholders’ environmental protection activities. The social strategy asked about consumer protection, improvement of the working environment, and win-win activities with partner firms (or competitors). Regarding governance strategy, the survey asked about process design to guarantee shareholder rights, continuous monitoring through an independent audit team, and reflecting stakeholders’ opinions. Each item was rated on a five-point Likert scale ranging from 1 (strongly disagree) to 5 (strongly agree).

This study used three control variables: firm size, industry type, and externalities. It measured firm size as a natural log of the firm’s total assets (in millions of Korean won) for 2020. This study controlled for size because larger firms have access to more or better capabilities than smaller firms, while smaller firms may have more flexibility and the ability to develop DCs more quickly. It measured the industry type based on data submitted by firms to the KRX. This study assigned a dummy variable to each firm based on dominant industry types: processing and manufacturing (MFG), which included 32 firms; sales and service (SVC), which included 34 firms; and 12 firms in other industries such as utilities, energy, chemicals, and transportation (Khanna and Rivkin, 2001; Kriauciunas and Kale, 2006). We used measures for the other industries as control variables with regard to manufacturing and industry, sales, and service. This study also included externalities in the models as a separate measure for control purposes because social concern may influence firm performance (Leong and Yang, 2020).

### Common Method Bias

In this study, the dependent and independent variables were subjectively measured by the same person at the same time. In this case, the answer itself might contain the respondent’s bias, which implies the possibility or risk of common method bias. Therefore, we verified whether standard method bias applied or not by performing one-factor analysis before conducting a full-scale statistical analysis.

According to Podsakoff et al. (2003), “One of the most widely used techniques that have been used by researchers to address the issue of common method bias is what has come to be called Harman’s one-factor (or single-factor) test” (p. 889). We entered all variables measured subjectively by the respondents into this testing method. The results showed that four factors were divided, and the largest factor was 43.34%, which suggests that common method bias was not a concern in this study. According to Podsakoff et al. (2003), the presence of a substantial number of common methods should be suspected in cases where (1) a single factor emerges from the factor analysis or (2) one largest factor accounts for the majority of the covariance among the measures (i.e., more than 50%).

We used externalities as a marker variable. Bootstrapping for the path coefficient and significance verification confirmed that a marker variable was not significant with all variables. In addition, it was proven that the path coefficient is greater before a marker variable is controlled for (see Table 1). This result indicates that common method bias is not a major problem in our data.

**Table 1:** Marker variable analysis results.

Marker control	Path	Original sample	Sample mean	Standard deviation	*T*	Value of *p*
Before	Absorptive capability → SMP	0.409	0.414	0.159	2.573	^**^
	Absorptive capability → ESG strategy	0.312	0.313	0.113	2.755	^**^
	Adaptive capability → SMP	−0.342	−0.366	0.178	1.916	0.056
	Adaptive capability → ESG strategy	0.531	0.540	0.109	4.863	^***^
	ESG strategy → SMP	0.581	0.607	0.159	3.662	^***^
After	Absorptive capability → SMP	0.372	0.357	0.186	2.003	^*^
	Absorptive capability → ESG strategy	0.311	0.316	0.112	2.789	^**^
	Adaptive capability → SMP	−0.323	−0.343	0.180	1.793	0.074
	Adaptive capability → ESG strategy	0.531	0.530	0.111	4.779	^***^
	ESG strategy → SMP	0.588	0.619	0.173	3.399	^***^
	Marker 1 → Absorptive capability	0.216	0.221	0.123	1.757	0.080
	Marker 2 → Adaptive capability	0.123	0.124	0.155	0.789	0.430
	Marker 3 → ESG strategy	0.004	0.004	0.084	0.044	0.965
	Marker 4 → SMP	0.075	0.087	0.112	0.616	0.538

^*^p < 0.05, ^**^p < 0.01, ^***^p < 0.001.

## Analyses and Results

### Analysis Method

We applied the partial least squares structural equation model (PLS-SEM), which is considered suitable for complex path models, and it has the advantage of being relatively free from strict and unrealistic assumptions (e.g., multivariate normality) and sample size (Hair et al., 2017). In general, the PLS-SEM focuses on predictive and exploratory analysis, compared to the covariance base SEM (Hair et al., 2018).

This study discusses the ESG strategy and DCs to achieve a firm’s SMP. There were relatively few prior studies that were not systematized; therefore, a strong attribute of the research is selection and analysis of measurement items. In particular, to consider sustainable management, analysis should be conducted from an integrated perspective that connects the firm’s capabilities and ESG strategy. For this reason, we decided that using PLS-SEM is more effective for stably estimating parameters and examining integrated causal relationships.

### Construct Validity

The validity was verified by analyzing the measurement model and the structural model. Measurement model analysis was verified in the following order: Cronbach’s alpha, multicollinearity, convergent validity, and discriminant validity. Cronbach’s alpha coefficient of all constituent concepts was 0.70 or higher (0.882 < all alpha coefficients <0.932). Multicollinearity is evaluated by the variance inflation factor (VIF), and in this study, the VIF values of all measured variables were less than 5 (2.254 < all VIF values <4.659), which confirmed there was no problem with multicollinearity. Convergent validity uses factor weights, outer loads, and average variance extracted (AVE). As shown in Table 2, the factor weights and factor loadings of all variables were significant, and the AVE values were greater than 0.50 for all constructs (0.597 < all AVE values <0.890), which provides strong evidence of convergent validity. Discriminant validity was evaluated by comparing the AVE estimates for each construct with the square of the parameter estimates between two constructs. According to Fornell and Larcker (1981), discriminant validity is achieved if the AVE of each construct exceeds the square of the standardized correlations between the two constructs. All AVE estimates were greater than the squared correlations between all constructs. Thus, multicollinearity, convergent validity, and discriminant validity were established (see Tables 2 and 3).

**Table 2:** Analysis results from measurement model.

Variable	Indicators	Cronbach’s *α*	VIF	Outer weights	Outer loadings	AVE
Absorptive capability	Ab1	0.931	2.304	0.171	0.802	0.674
	Ab2		2.617	0.144	0.787	
	Ab3		3.516	0.111	0.786	
	Ab4		3.025	0.126	0.731	
	Ab5		4.107	0.167	0.874	
	Ab6		3.442	0.158	0.808	
	Ab7		4.388	0.163	0.895	
	Ab8		4.140	0.173	0.873	
Adaptive capability	Ad1	0.921	4.334	0.202	0.891	0.718
	Ad2		2.623	0.156	0.773	
	Ad3		4.128	0.218	0.876	
	Ad4		2.349	0.167	0.811	
	Ad5		3.935	0.218	0.863	
	Ad6		3.808	0.214	0.863	
ESG strategy	ESG1	0.932	3.674	0.096	0.670	0.651
	ESG2		3.780	0.113	0.700	
	ESG3		3.110	0.147	0.765	
	ESG4		4.295	0.154	0.901	
	ESG5		3.636	0.140	0.838	
	ESG6		3.704	0.145	0.840	
	ESG7		4.190	0.130	0.836	
	ESG8		4.581	0.143	0.851	
	ESG9		4.659	0.163	0.831	
SMP	SMP1	0.903	2.611	0.160	0.753	0.597
	SMP2		2.254	0.170	0.677	
	SMP3		3.310	0.132	0.789	
	SMP4		2.580	0.163	0.775	
	SMP5		3.283	0.206	0.846	
	SMP6		4.190	0.166	0.774	
	SMP7		4.581	0.155	0.750	
	SMP8		4.659	0.143	0.808	
Externalities	Ex1	0.882	2.648	0.644	0.968	0.890
	Ex2		2.648	0.411	0.918	

**Table 3:** Fornell–Larcker criterion.

Variable	1	2	3	4	5	6	7	8
1. Firm size	**–**							
2. Type (MFG)	−0.003	**–**						
3. Type (SVC)	0.012	−0.713	**–**					
4. Externalities	0.143	0.202	0.046	**0.943**				
5. Absorptive capability	0.167	−0.111	0.180	0.215	**0.821**			
6. Adaptive capability	0.153	−0.119	0.165	0.094	0.840	**0.847**		
7. ESG strategy	0.131	−0.196	0.211	0.099	0.759	0.791	**0.807**	
8. SMP	0.206	−0.131	0.188	0.178	0.567	0.467	0.606	**0.773**

The PLS-SEM can evaluate the structural model with the coefficient of determination (R^2^) and predictive relevance (Q^2^). The coefficient of determination from building an ESG strategy was 0.656, and SMP was 0.436. Moreover, blindfolding was performed to examine the predictive relevance of endogenous reflection indicators and single-item scales. Q^2^ was obtained from the sum of squares of observations (SSO) for SMP and the sum of squares for predictive error (SSE). Looking at the analysis results, the Q^2^ of SMP was 0.237 with a value of 0 or higher, and hence, the Q^2^ of the structural model for endogenous potential variables exists (Sarstedt et al., 2016, see Table 4).

**Table 4:** Predictive relevance (Q2) results.

Variable	SSO	SSE	Q2
Firm size	78.000	78.000	
Type (MFG)	78.000	78.000	
Type (SVC)	78.000	78.000	
Externalities	156.000	156.000	
Absorptive capability	624.000	624.000	
Adaptive capability	468.000	468.000	
ESG strategy	702.000	410.349	0.415
SMP	624.000	475.923	0.237

### Hypothesis Testing

**Figure 2:**
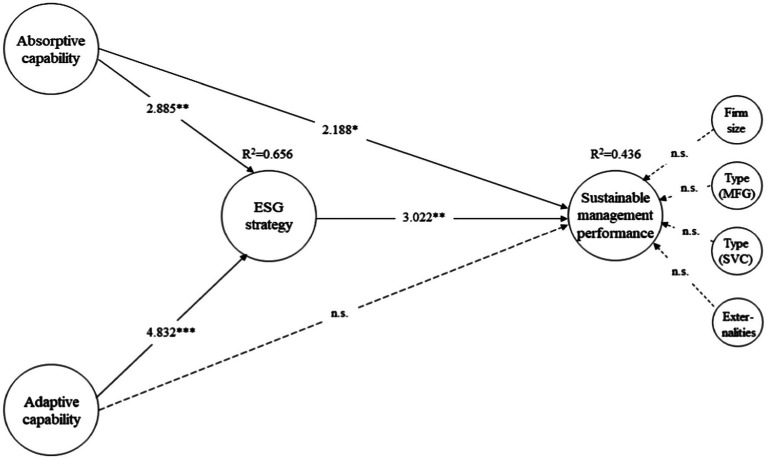
Estimated results of a structural equation analysis. Non-significant paths are shown with a dotted line; n.s. = not significant. ^*^p < 0.05, ^**^p < 0.01, ^***^p < 0.001.

Figure 2 presents the results of the structural equation model. The results show that the absorptive capability (*β* = 2.188, *p* < 0.05) had a positive relationship with SMP. However, the adaptive capability (*β* = 1.606, *p* = 0.109) had no significant relationship with SMP. These results support Hypothesis 1–1, but not Hypothesis 1–2. Furthermore, we examined the mediating effects of the ESG strategy between the DCs (absorption and adaptation capabilities) and SMP. As a result of bootstrapping, absorptive capability (*β* = 2.885, *p* < 0.01) and adaptive capability (*β* = 4.832, *p* < 0.001) were analyzed as having a positive effect on the ESG strategy. The path from the ESG strategy to SMP was also positive and significant (*β* = 3.022, *p* < 0.01). In addition, the indirect path (DCs → ESG strategy → SMP) was also analyzed as significant, and it is possible to determine whether there is partial mediation. More importantly, when the mediating variable (i.e., the ESG strategy) was included in the model, the R^2^ of SMP further increased from 0.372 to 0.436. Overall, these results demonstrate that ESG strategy implementation plays an important role in mediating between DCs and SMP. Thereby Hypotheses 2 is supported. Meanwhile, investigating whether control variables such as firm size, firm type, and externalities affected SMP did not show statistically valid results.

## Discussion and Conclusion

In this study, we theorized and addressed two central research questions: (1) What capabilities and structures are required to achieve a firm’s SMP? (2) How does the ESG strategy improve this performance? We developed a series of hypotheses by adopting a DCs perspective to explore the capabilities and strategies needed to create a firm’s SMP. According to previous studies, DCs is generally a key factor that closely affects achieving a competitive advantage by improving a firm’s performance. In particular, we argued that SMP can be achieved when implementing an ESG strategy based on DCs. Given the recent changes and uncertainties surrounding the business environment, we suggested that implementing strategies that meet social trends, such as having an ESG strategy, can serve as an essential mechanism for a firm’s economic (or financial) performance as well as social and environmental performance.

The DCs perspective has long highlighted that a firm’s competitive advantage is driven by the capabilities built into the process of responding to environmental changes. Building upon this analytical and theoretical underpinning, we linked two types of capabilities (absorptive capability and adaptive capability) to respond to the rapidly changing business environment, and business performance is important to a firm’s sustainability. Moreover, we argued that firms should implement ESG strategies in order to achieve the sustainable value required by modern society. An empirical test on a sample of firms that implemented an ESG strategy in Korea supports some of our hypotheses. We obtained partially significant statistical effectiveness from the hypothesis that DCs (i.e., absorption and adaptation capabilities) respond to a changing environment and directly or indirectly affect SMP. In addition, we found that if the firms implemented an ESG strategy through DCs, they were more likely to achieve SMP. This evidence suggests that a firm’s ESG strategy implementation has a vital role in promoting competitive advantage based on DCs. Our empirical findings demonstrate that firms’ ESG implementation efforts can help strengthen their competitive position in terms of sustainability. Meanwhile, The Korean government is enacting various norms from an institutional perspective to facilitate ESG implementation in firms. This can act as regulatory pressure on firms, leading to an increase in the cost of regulatory compliance. Our research results can suggest a direction for firms to redefine their capabilities and develop ESG strategies in terms of preemptive response.

Our study offers important theoretical contributions to DCs research. First, our study contributes to the literature on a firm’s sustainable competitive advantage creation. Specifically, we adopted a DCs perspective to show the importance of a firm’s capabilities and its strategy in affecting SMP. More importantly, this study contributes to the competitive advantage literature by providing new insights into the role of an ESG strategy in creating SMP. We expand corporate sustainable development research through strategic frameworks based on two types of DCs. These two DCs seem important in encouraging firms to implement ESG strategies. In addition, we contribute to the literature theoretically by identifying and capturing the social and environmental performance required for firms to improve sustainability. Despite great efforts by prior scholars on this issue, they generally focused on achieving results in terms of finance and innovation, ignoring the possibility that competitive advantage may influence social and environmental performance. We thus advance the understanding of the literature on sustainability and DCs by linking capabilities with sustainability, and by capturing and measuring effects at the firm level. By integrating ESG literature with the DCs perspective, we highlighted ESG strategy implementation based on DCs in achieving sustainable performance. To the best of our knowledge, our study is one of the first to examine how the various types of DCs shape an ESG strategy and the benefits such an ESG strategy provides to a firm’s SMP.

Our study also provides important implications for a practical audience. This study shows that ESG strategy implementation can positively contribute to enhancing a firm’s SMP. Undoubtedly, firms can benefit from implementing ESG activities when operating their businesses. Our study particularly suggests that firms can improve their SMP in terms of social and environmental performance as well as financial performance by actively responding to changing environments through implementation of ESG strategies based on DCs. Furthermore, managers of firms should be aware that DCs might not directly contribute to SMP. Therefore, most importantly, firms should recognize the importance of effectively implementing ESG strategies through DCs.

Like all research, this study has limitations. First, our sample only consists of firms in Korea, which may raise concerns about generalizing on the effectiveness of ESG strategies. Future research can verify the generalization of our frameworks and empirical results by expanding our study with samples of firms operating in other countries that promote ESG implementation. Second, we acknowledge that this study incorporated only a limited set of DCs and outcome variables into the analysis. Additional variables and conditions should be considered when exploring the forces to form a firm’s ESG strategy and its implications. Previous research has emphasized the importance of a firm’s various dynamic capabilities in forming suitable strategic choices (e.g., Wang and Ahmed, 2004, 2007; Agarwal and Selen, 2013; Ringov, 2017). Therefore, future research is recommended to investigate whether and how firms’ various DCs can form ESG strategies differently and how these firms can achieve successful SMP by using ESG strategies. In the same line, the detailed process (e.g., sensing, integrating, and reconfiguration) of constituting DCs can be considered. Third, although we argue that the issue of reverse causality is of less concern in our study, we acknowledge that the problem may still be found in this type of cross-sectional research. As well, data constraints do not adopt longitudinal data or experimental methods to guard against the possibility of having a reverse-causality effect that biases the results. Moreover, due to data unavailability, we cannot capture in this study the possible dynamic nature of ESG strategy forces. Future research can further capture the dynamic effect of ESG strategies using longitudinal data. Studies can also explore how various DCs respond to environmental changes during implementation, and how ESG strategies contribute to a firm’s strategic response and various types of performance. This is another potential future research avenue. Finally, as in a recent study by Bhandari et al. (2022), empirical analysis using ESG disclosure data emerged. This study used secondary data through a survey, which can be limiting in terms of ensuring the legitimacy of the data.

Meanwhile, using ESG disclosure could have been even better because this information is publicly out with some evidence at least (Christensen et al., 2013; Winkler et al., 2020). Using ESG disclosure data is useful to ensure the legitimacy of the methodology. For reference, in Korea, each firm discloses an ESG implementation report annually. However, these reports have no standards, and there are limitations in using them as analysis data because the contents are different for each firm. Of course, the Korean government is gradually promoting the disclosure of such ESG-related information, which is expected to be supplemented in future research issue.

In conclusion, our study offers a nuanced understanding of how a firm’s DCs may influence its ESG strategy, and how they can be used to achieve sustainable performance in Korean context (institutional and social). We believe our findings provide valuable insights for improving a firm’s sustainability by enabling scholars and practitioners to deepen their understanding of the importance of ESG strategic management.

## Data Availability Statement

The original contributions presented in the study are included in the article/supplementary material, further inquiries can be directed to the corresponding author/s.

## Author Contributions

YL and ML contributed to the conceptualization, methodology, investigation and writing – original draft. ML and JJ performed research model, data collection, data curation and formal analysis. YL, ML, and JJ participated in the manuscript revision, review, editing and validation. All authors have read and approved the final manuscript.

## Conflict of Interest

The authors declare that the research was conducted in the absence of any commercial or financial relationships that could be construed as a potential conflict of interest.

## Publisher’s Note

All claims expressed in this article are solely those of the authors and do not necessarily represent those of their affiliated organizations, or those of the publisher, the editors and the reviewers. Any product that may be evaluated in this article, or claim that may be made by its manufacturer, is not guaranteed or endorsed by the publisher.

## Footnotes

^1^The CEO of the U.S. Business Roundtable deleted the phrase “maximizing shareholder value” from the firm’s purpose, and argued that firms should also invest in employees and provide value to customers (*New York Times*, 2019.8.19).

## Appendix 1 Previous Literature on Dynamic Capabilities

**Table:**

Performance measures	DCs → Performance	Antecedents to DC → DC (mediator or intermediate outcomes) → Performance	DC → Mediator/intermediate variable → Performance
Innovation performance	Agarwal and Selen (2013), Cheng et al. (2014), Chiu et al. (2016), Falasca et al. (2017)	Zheng et al. (2011), Cheng et al. (2014), Han and Li (2015), Wu et al. (2016), Falasca et al. (2017)	
Economic/financial performance	Wu (2007), Wilden et al. (2013), Wang et al. (2015), Ringov (2017), Fainshmidt et al. (2017), Girod and Whittington (2017), Konwar et al. (2017), Kumar et al. (2018)	Wu (2007), Lin and Wu (2014), Piening and Salge (2015), Wang et al. (2015), Fainshmidt et al. (2017), Ko and Liu (2017)	Liu and Hsu (2011), Ju et al. (2016), Vickery et al. (2013), Mu (2017)
Operation/process performance	Drnevich and Kriauciunas (2011), Wilhelm et al. (2015), Ju et al. (2016), Kumar et al. (2018)	Blome et al. (2013)	Ju et al. (2016)
Organizational/firm performance	Hung et al. (2010), Chien and Tsai (2012), Hsu and Wang (2012), Jiang et al. (2015), Peng and Lin (2017)	Hung et al. (2010), Chien and Tsai (2012), Hsu and Wang (2012), Sarkar et al. (2016), Peng and Lin (2017), Wamba et al. (2017)	Vanpoucke et al. (2014), Jiang et al. (2015), Wilden and Gudergan (2015, 2017), Lee and Rha (2016), Battisti and Deakins (2017), Mu (2017)
Competitive advantage	Li and Liu (2014), Schilke (2014), Wu (2010), Gelhard et al. (2016), Lin and Chen (2017)		Lin and Chen (2017), Mikalef and Pateli (2017), Ferreira et al. (2020)
Export performance	Monteiro et al. (2019)	Villar et al. (2014), Monteiro et al. (2019)	
International performance	Pinho and Prange (2016), Swoboda and Olejnik (2016)	Monferrer et al. (2015), Pinho and Prange (2016), Swoboda and Olejnik (2016)	
Portfolio performance	Mitrega and Pfajfar (2015)	Mitrega and Pfajfar (2015), Hermano and Martín-Cruz (2016)	
Product development performance	Chen and Chang (2013)	Zhang and Wu (2017)	Chen and Chang (2013), Cai et al. (2014), Mu (2017)
Project performance		Hermano and Martín-Cruz (2016)	
Response performance	Karimi and Walter (2015)		Karimi and Walter (2015)

## Appendix 2 Variables and Measures

**Table:**

Variable	Measurement (5-point scale)	References
SMP	[SMP1] What is your firm’s net profit growth level compared to its competitors over the previous five years? [SMP2] What is the level of increase in your firm’s market share compared to its competitors over the previous five years? [SMP3] What is the level of contribution to alleviating your (individual, regional, and national) inequality compared to competitors over the previous five years? [SMP4] What is the level of contribution to strengthening your firm’s social safety compared to your competitors over the previous five years? [SMP5] What is the level of contribution of your firm to solving overall social problems compared to its competitors over the previous five years? [SMP6] What is your firm’s level of reduction in carbon emissions compared to its competitors over the previous five years? [SMP7] What is the level of reduction in your firm’s resource (energy) usage compared to its competitors over the previous five years? [SMP8] What is your firm’s level of overall environmental performance compared to its competitors over the previous five years?	Lumpkin and Dess, 2006; Panigyrakis and Theodoridis, 2007; Wang and Ahmed, 2007; Zhu et al., 2008; Adebanjo et al., 2016; Wu et al., 2016; Abdul-Rashid et al., 2017; Inman and Green, 2018
Absorptive capability	[Ab1] Your employees have sufficient learning ability to acquire external knowledge. [Ab2] Your firm actively participates in activities to acquire external knowledge. [Ab3] Your firm implements externally acquired knowledge (or information) in the organization. [Ab4] Your firm uses a variety of processes within the organization to capture external knowledge (or information). [Ab5] Your firm converts (socializes) the acquired knowledge (or information) appropriately for your organization. [Ab6] Your firm combines the acquired knowledge (or information) with the organization and seeks transformation. [Ab7] Your firm links new knowledge (or information) to work. [Ab8] Your firm improves problem-solving functions by applying new knowledge (or information).	García-Morales et al., 2008
Adaptive capability	[Ad1] Your firm monitors customers and competitors to respond quickly to changing market conditions. [Ad2] Your firm allocates resources to marketing activities to respond quickly to changing market conditions. [Ad3] Your firm has strategies and processes to respond to the changing market environment quickly. [Ad4] Your firm encourages employees to challenge outdated traditions and practices. [Ad5] Your firm has a system (or process) that can quickly redefine itself according to changes in business priorities. [Ad6] Your firm has a management system (or process) that can determine whether you are responding quickly to market changes.	Gibson and Birkinshaw, 2004; Wang and Ahmed, 2004
ESG strategy	[ESG1] Your firm is establishing environmental management strategies and action plans. [ESG2] Your firm manages its environmental performance through evaluation and audit systems. [ESG3] Your firm actively supports the environmental protection activities of stakeholders. [ESG4] Your firm actively participates in consumer protection. [ESG5] Your firm actively participates in improving the working environment. [ESG6] Your firm actively participates in mutual life with its partners (or competitors). [ESG7] Your firm is building a process to guarantee shareholders’ rights. [ESG8] Your firm has established independent audit organizations inside and outside and monitors them at all times. [ESG9] Your firm listens to opinions from stakeholders and markets and reflects them in management.	Developed for this study based on the K-ESG index.
Externalities	[Ex1] What is the community’s level of interest in implementing ESG strategies? [Ex2] What is the level of customer or public expectations for ESG performance?	Leong and Yang, 2020
